# Erythrocytes as a Model for Heavy Metal-Related Vascular Dysfunction: The Protective Effect of Dietary Components

**DOI:** 10.3390/ijms22126604

**Published:** 2021-06-20

**Authors:** Rosaria Notariale, Rosmara Infantino, Enza Palazzo, Caterina Manna

**Affiliations:** 1 Department of Precision Medicine, School of Medicine, University of Campania “Luigi Vanvitelli”, 80138 Naples, Italy; notarialer@gmail.com; 2 Department of Experimental Medicine, Division of Pharmacology, University of Campania “Luigi Vanvitelli”, 80138 Naples, Italy; rosmainfantino@gmail.com (R.I.); enza.palazzo@unicampania.it (E.P.)

**Keywords:** cardiovascular diseases, endothelium, erythrocytes, heavy metals, mercury, nutrition, oxidative stress, polyphenol

## Abstract

Heavy metals are toxic environmental pollutants associated with severe ecological and human health risks. Among them is mercury (Hg), widespread in air, soil, and water, due to its peculiar geo-biochemical cycle. The clinical consequences of Hg exposure include neurotoxicity and nephrotoxicity. Furthermore, increased risk for cardiovascular diseases is also reported due to a direct effect on cardiovascular tissues, including endothelial cells, recently identified as important targets for the harmful action of heavy metals. In this review, we will discuss the rationale for the potential use of erythrocytes as a surrogate model to study Hg-related toxicity on the cardiovascular system. The toxic effects of Hg on erythrocytes have been amply investigated in the last few years. Among the observed alterations, phosphatidylserine exposure has been proposed as an underlying mechanism responsible for Hg-induced increased proatherogenic and prothrombotic activity of these cells. Furthermore, following Hg-exposure, a decrease in NOS activity has also been reported, with consequent lowering of NO bioavailability, thus impairing endothelial function. An additional mechanism that may induce a decrease in NO availability is the generation of an oxidative microenvironment. Finally, considering that chronic Hg exposure mainly occurs through contaminated foods, the protective effect of dietary components is also discussed.

## 1. Introduction

Heavy metals are a group of ubiquitous and non-biodegradable pollutants widely distributed in the environment. Heavy metal pollution of the environment results not only from natural sources such as volcanic activity and fossil fuels, but also from numerous agricultural, medical, industrial, and technological human activities [1,2]. Exposure to heavy metals in humans occurs mainly from contamination of water, soil, and food. The exposure to low levels of heavy metals in the general population is widely recognized while massive exposure affects specific subpopulations under certain circumstances, especially occupational ones. Exposure to heavy metals is associated with both short-term adverse effects and long-term diseases such as cancers, immune system dysfunctions, neurocognitive impairments, behavioral abnormalities, hormonal and metabolic dysregulation, and specific organ damage [3,4,5].

It should be emphasized that living systems interact in the environment not only with a single heavy metal [6,7,8,9] but more often with a cocktail of compounds that can have synergistic adverse effects on the organism [10,11,12,13]. Preclinical and clinical studies have investigated the toxicity of mixtures of the main heavy metals [14] that pollute the environment such as lead, mercury (Hg), chromium, cadmium, and arsenic on various organs, systems, or conditions [15,16,17,18,19]. A close association has been reported with immune system dysfunctions [20], bladder cancer [21], neurotoxicity [22,23], and embryogenesis defects [24]. Although the toxicity mechanisms associated with heavy metals are still poorly understood, common mechanisms underlying their toxicity both as single entities or mixtures have been identified [25,26,27,28]. Reactive oxygen species (ROS) production and oxidative stress (OS) are major mechanisms leading to protein alteration, lipid peroxidation, and DNA damage [29,30,31]. Among the various heavy metals, Hg has drawn particular attention, as it is a major environmental pollutant whose levels have been continuously increasing in recent decades due to its increased release in the environment by industrial waste or fuel extraction procedures [32]. Noteworthy is the fact that Hg has been ranked among the 10 chemicals of greatest concern for human health risk by the World Health Organization (WHO) [33].

## 2. Mercury Exposure and Toxicity

The global burden of Hg toxicity represents a serious public health concern worldwide. Hg exists in the ecosystem in elementary, inorganic, and organic forms. Elemental mercury (Hg^0^) is liquid and volatile at room temperature. This metal also exists as mercurous (Hg^+^) and mercuric (Hg^2+^) cation and can form both inorganic and organic compounds, methylmercury (MeHg) being the most widespread organic compound in the environment and the most important biologically [33,34]. MeHg is readily absorbed by the human body, which does not have an active excretion system for this element.

**Figure 1:**
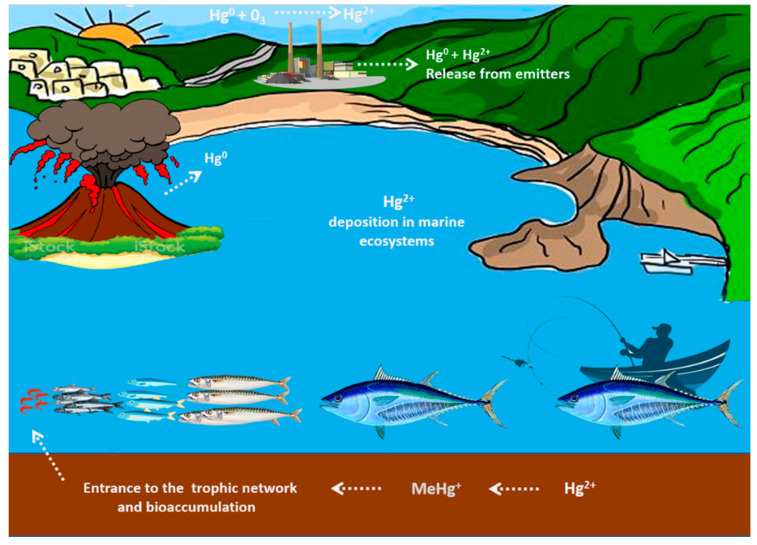
The Hg biogeochemical cycle. Hg pollution of the environment mainly results from natural sources such as volcanic activity and fossil fuels as well as human industrial activities. Hg exists in the ecosystem in elementary, inorganic, and organic forms. These different species can be environmentally as well as biologically converted into each other in soil, air and especially water. In aquatic sediments, inorganic Hg is converted into its organic forms, mainly methylmercury, by microorganisms. This form enters the trophic network via plankton and it accumulates in the passage through small to carnivorous fish at the top of the food chain, via the process of biomagnification, reaching humans through contaminated fish.

The different Hg molecular species can be environmentally as well as biologically converted into each other in soil, water, and air, due to their peculiar geo-biochemical cycle (Figure 1) as well as in our body. In aquatic sediments, a small fraction of Hg^2+^ is converted to organic forms by several kinds of anaerobic microorganisms which [35] enter the food chain through the contamination of fish and shellfish. Here Hg undergoes a process of bioaccumulation and biomagnification along the aquatic food chain until it reaches humans [36]. Conversely, Hg inhaled in elemental form or introduced orally in the form of MeHg, undergoes oxidation by catalase or demethylation in the liver [37,38]. Of the various sources of mercury contamination, food is certainly the most important in the general population. Other sources of contact with mercury affecting a wider range of individuals may be thermometers, batteries and some types of vaccines [39], which are responsible for possible accidental intoxications [40]. Although Hg^0^ and inorganic compounds are potentially dangerous to human health, exposure to these forms is generally limited to individual occupational exposure. However, Hg^0^ can be released from dental amalgams, thus representing one of the main sources of chronic exposure to low levels of Hg in humans [41,42].

The molecular mechanisms underlying Hg-induced cytotoxicity are complex, with likely contributions from genetic susceptibility [43] and exposure to the different molecular forms of this metal.

This metal, endowed with a high binding capacity to the sulfhydryl (SH) group, reacts with small molecular weight thiols, including alpha lipoic acid, and glutathione (GSH), thus impairing key metabolic pathways as well as the antioxidant defense system [44,45]. In this respect, Hg binding to selenium-containing proteins inhibits enzymes such as glutathione peroxidase and hinders the restoration of intracellular redox balance, thus worsening OS [46]. Interestingly, the genetic predisposition to the development of antioxidant system dysfunctions is associated with polymorphisms that determine a greater susceptibility to MeHg toxicity [43,47,48]. Specific pathways such as that of thioredoxin are also inhibited by Hg [49,50,51].

In addition, by reacting with crucial cysteine (Cys) residues, Hg may interact with cellular proteins, thus altering and inhibiting their enzymatic and structural functions and potentially leading to severe dysfunction in cellular activities [52]. In particular, Hg inhibitory activity on the glycolytic enzymes hexokinase and phosphofructokinase has been reported [53]. Also, membrane proteins are modified by Hg, including tubulin [54] and erythrocyte (RBC) band 3 and 4.1 and 4.2 proteins [55].

An additional mechanism of Hg toxicity is the induction of mitochondrial dysfunction affecting the activity of F1-F0-ATPase [56], causing oxidative phosphorylation and electron transport defects. This can in turn increase ROS production [57] and redox homeostasis disruption [58]. Furthermore, the carnitine/acylcarnitine transporter has been identified as a possible target of Hg toxicity in mitochondria [59].

**Figure 2:**
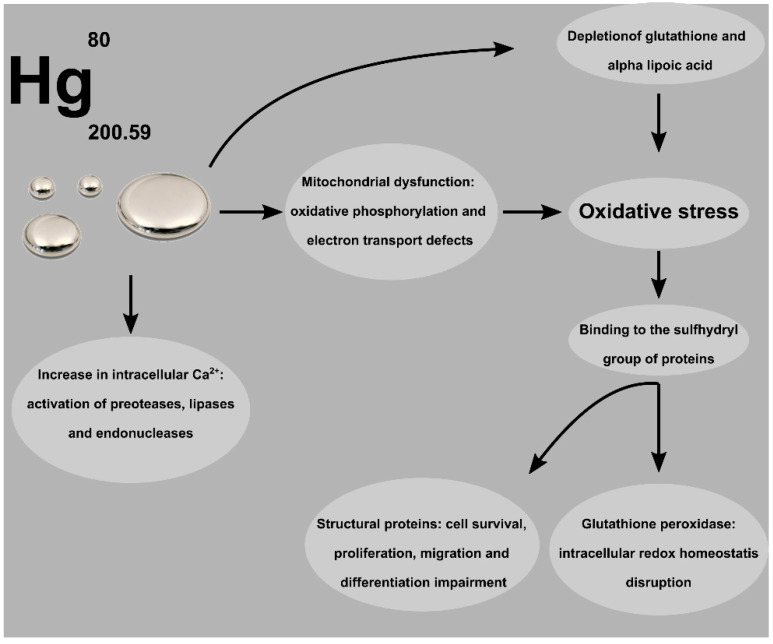
Mechanisms of toxicity associated with Hg exposure, most of which lead to oxidative stress.

Mercury toxicity is also associated with an increase in intracellular Ca^2+^ due to both an increase in the influx of Ca^2+^ from the extracellular environment and an increase in the mobilization of intracellular reserves [60]. This effect on calcium homeostasis, associated with the effect of calcium increase due to OS, is responsible for the activation of proteases, lipases, and endonucleases. Figure 2 shows a simplified diagram of the main mechanisms of Hg toxicity.

## 3. Acute Poisoning and Long-Term Toxicity

The health risk for mankind following Hg exposure has been well documented by a long series of epidemiological and experimental studies. Hg poisoning is usually caused by ingestion or inhalation of its vapors. Acute mercury poisoning can cause various disorders, among which acrodynia is typical [61]. Other manifestations of mercury poisoning are pain in the extremities, neurological disorders such as ataxia, and confusional states (cardiovascular effects such as tachycardia and hypertension), and other non-specific symptoms such as fever, flu-like syndromes, skin rash, nausea, vomiting, and diarrhea [40,62,63]. A delay in treatment can cause serious consequences, such as pneumonitis, renal tubular necrosis and neurological dysfunction [64].

The intoxication by the organic compound MeHg has been associated with the tragedy of Minamata (Japan) in the 50 s. The discharge of this compound in the gulf adjacent to the city caused a serious intoxication of the entire population [65]. The intoxication caused mental disorders, paralysis, coma, and death within a few weeks of the first symptoms [66]. A congenital form of the disease can be transmitted to the fetus during pregnancy causing delayed psychomotor development in infants [67]. This tragic event however has led to a deeper understanding of the mechanisms of MeHg toxicity. Although the mechanism underlying the tissue-selective vulnerability of the central nervous system (CNS) by MeHg remains to be elucidated, post-mortem studies of the above-mentioned mercury intoxication have shown petechial hemorrhages and cerebral edema, suggesting damage to the blood-brain barrier or a general involvement of vascular dysfunction [58,68]. These observations have been confirmed by the finding of a long-term increased incidence of hypertension and myocardial ischemia in the population living in the neighboring areas of Minamata [69].

Numerous studies have shown that long-term exposure to even small amounts of MeHg also affects endothelial function [70,71]. Nowadays, it is widely accepted that Hg exposure may be a risk factor for cerebrovascular and cardiovascular diseases (CVD), even without typical symptoms of Hg intoxication [58,72].

## 4. Mercury and Endothelial Dysfunction

The correlation between Hg exposure and endothelial dysfunction was confirmed after follow-up studies on severe cases of poisoning, the first being that of Minamata in Japan, which highlighted various cardiovascular anomalies [58,73]. Endothelial cells are very vulnerable to Hg-induced OS caused either by increasing the production of oxidative agents or by inducing a decrease in antioxidant activity [71,74]. The increase of OS induces, via various pathways, endothelial inflammation first and then endothelial dysfunction, ref. [73,75] followed by the development of atherosclerosis, thrombophilia diathesis, and risk of ischemic phenomena through vessel obstruction or vasospastic events [76]. Polymorphisms in genes involved in antioxidant activity in the populations exposed to mercury have been shown to be associated with MeHg retention and increased risk of myocardial infarction [76,77]. Another important mechanism of Hg toxicity on the endothelium is the reduction of nitric oxide (NO) levels [78]. NO is an important cellular signaling molecule, which plays a crucial role in endothelium physiology, synthesized from the amino acid L-arginine by nitric oxide synthase (NOS) isoforms, mainly in endothelial cells [79].

Reduced NO bioavailability, together with the Hg-induced increase in OS, predisposes to CVD, such as carotid atherosclerosis, myocardial infarction, coronary heart disease and hypertension [80,81]. Exposure to low concentrations of Hg in the rat tail artery caused an increase in vascular resistance, mediated by an increase in ROS production, the production of vasoconstrictor mediators and the reduction in the bioavailability of NO [82]. In particular, superoxide anion inactivates NO [83], a potent vasodilator, causing in turn vasoconstriction of arteries [84]. Acute Hg exposure also produced vasoconstriction in rat aortic rings by increasing ROS (via NADPH oxidase) and inhibition of NO production [85]. The chronic exposure to low concentrations of Hg produced similar effects: increased production of ROS, reduced bioavailability of NO and endothelial dysfunction in the mesenteric, coronary, and basilar arteries [82,86]. Hg-induced endothelial damage also involves vasoconstrictor proteinoids derived from the COX-2 pathway and plasma angiotensin-converting enzyme (ACE) [87]. Concomitant mechanisms associated with Hg-induced toxicity are decreased proliferation and migration of endothelial cells, activation of coagulation pathways by promoting platelet aggregation and activation of factor XIII [58]. Hg poisoning is thus associated with the risk of atherothrombotic diseases, including hypertension, cerebrovascular disease, acute myocardial infarction, and renal dysfunction [80,88,89,90,91]. The main organs to be damaged by endothelial dysfunction are therefore the heart, kidneys, and brain [78,80,91]. Vascular dysfunction is also related to the development of hypertension, which exponentially increases the aforementioned risks [90].

## 5. Mercury and Hypertension

Besides the traditional risk factors associated with hypertension (overweight/obesity, sedentary lifestyle, cigarette smoke and excess salt intake) environmental exposures to heavy metals may also play an important role [92,93]. The most important cohort studies addressed towards the risk of hypertension from Hg exposure are those that were performed on miners or populations particularly exposed to this heavy metal such as Eskimos, inhabitants of the Minamata region, workers and fish consumers. It was observed that Hg miners showed a significant increase in systolic blood pressure which correlated with lipid peroxidation and total OS and an increased risk of developing hypertension of about 50% [94]. Other important studies have shown a close correlation between the Hg content in hair (a marker of Hg exposure) and the incidence of hypertension [95,96]. Other studies, in contrast, have correlated hypertension to the blood content of Hg, repeatedly finding a close association between high levels and hypertension [97,98]. In addition to hypertension, Hg exposure is also associated with a reduction in heart rate variability. The latter predisposes to arrhythmias, ventricular fibrillation, sudden cardiac death, angina, myocardial infarction, CVD, and cerebrovascular accidents [99].

One of the main mechanisms through which both acute and chronic Hg intoxication leads to hypertension is the binding of Hg to the sulfhydryl group of S-adenosyl methionine. This bond causes inactivation of the enzyme catecholamine-O-methyl transferase (COMT) which uses S-adenosyl-methionine as a cofactor. Since this enzyme operates the inactivation by O-methylation of adrenaline, noradrenaline, and dopamine [90]. Its inactivation causes an increase in circulating catecholamines and a syndrome like a pheochromocytoma crisis. This syndrome is characterized by malignant hypertension in acute intoxication and increased urinary excretion of catecholamines in chronic Hg intoxication. Another mechanism associated with hypertension is Hg toxicity with regard to the kidney. This metal concentrates in the renal tubules and glomeruli causing proteinuria and renal fibrosis and failure [100]. Renal dysfunction contributes to water and sodium retention and ultimately to hypertension [82,90,91]. In fact, an increased risk of renal failure of 55% was observed in Hg miners compared to the general population [91].

## 6. The Role of Erythrocytes in Hg-Induced Endothelium Dysfunction

In this context, it is important to highlight the proposed role of RBC in endothelial dysfunction and the risk of CVD. In fact, an increasing body of data has recently been accumulated on the role of these cells in both hemostasis and coagulation. Based on these findings, Weisel et Litvinov [101] defined RBC as “the forgotten actor” in endothelial dysfunction, reviewing how both quantitative and qualitative changes could affect bleeding or thrombosis [101]. Among the observed alterations, phosphatidylserine (PS) exposure at the outside leaflet of the cellular membrane has been proposed as a candidate hallmark of increased proatherogenic and prothrombotic activity of these cells. Specifically, PS exposure facilitates RBC adhesion to the endothelium, contributing to clot formation [102,103]. Furthermore, microvesicles (MVs) formation also leads to increased pro-coagulating events [104]. The key role of extracellular vesicles from RBC in health, coagulopathy and therapy has been recently reviewed [105].

Mammalian RBC are anucleate cells, deformable thanks to the shape of a biconcave disc, also lacking all the intracellular organelles, and thus representing a simplified model for metabolism studies. Moreover, they are deeply sensitive cells and important health markers because they have a highly specialized and organized cell membrane, which interacts with inflammatory oxidizing agents and mediators, leading to a variety of structural changes that readily signal an abnormal situation [106,107]. Late enucleation of erythroblasts in mammals is considered an advantage in the investigation of particular aspects of these cells as it defines a starting point of an average aging process of 120 days until their elimination. Accordingly, RBC have been identified for use as a cellular aging model [108] as well as being markers of OS-related pathologies, including CVD [106,108]. In fact, RBC are particularly advantageous for the study of OS caused by the high tension of oxygen and the highly toxic free radicals derived from it. Interestingly, being endowed with a powerful antioxidant defense machinery, these cells significantly contribute to other blood cells as well as endothelium from the oxidative insult. However, if RBC reach highly inflamed tissues, such as endothelium with atherosclerotic lesions, their behavior shifts from the physiological activity of scavenger to the harmful role of ROS generator, inducing an oxidative microenvironment, thus worsening the endothelial dysfunction. Therefore, the antioxidant status of RBC might represent a marker in CVD [106,109].

Similarly, RBC have been proposed as a model for pharmacological and toxicological studies which explore mechanisms underlying heavy metal toxicity, including Hg. The effect that toxic substances have on these cells is often omitted, underestimating the concept that blood is the major tissue in contact with exogenous molecules. Moreover, blood also acts as a vehicle of substances, which reach all tissues. As far as heavy metal toxicity is concerned, RBC are a preferential site for Hg accumulation, reaching in these cells a concentration 20 times higher than that found in plasma [110].

The direct toxicity effect of Hg on RBC has been amply investigated in the last few years, both in vivo and in vitro. In a recent review, Vianna et al. [111] discuss the available evidence on potential hematological effects of human mercury exposure, including lymphopenia, lymphocytosis, neutrophilia and basophilia. The possible occurrence of anemia associated with Hg exposure has also been reported [111,112], likely due to hemolysis [112,113,114], loss of blood from the direct effect on the gastrointestinal mucosa and apoptosis [112,114]. Similarly to apoptosis of nucleated cells, RBC may undergo programmed cell death, also called eryptosis [115,116]. Interestingly, both eryptosis and apoptosis of nucleated cells share the feature of externalization of PS [117]. This altered membrane asymmetry results in the erythrophagocytosis of aged RBC, thus regulating the life span of circulating mature cells as well as removal of stressed/damaged RBC in pathological situations [118,119].

**Figure 3:**
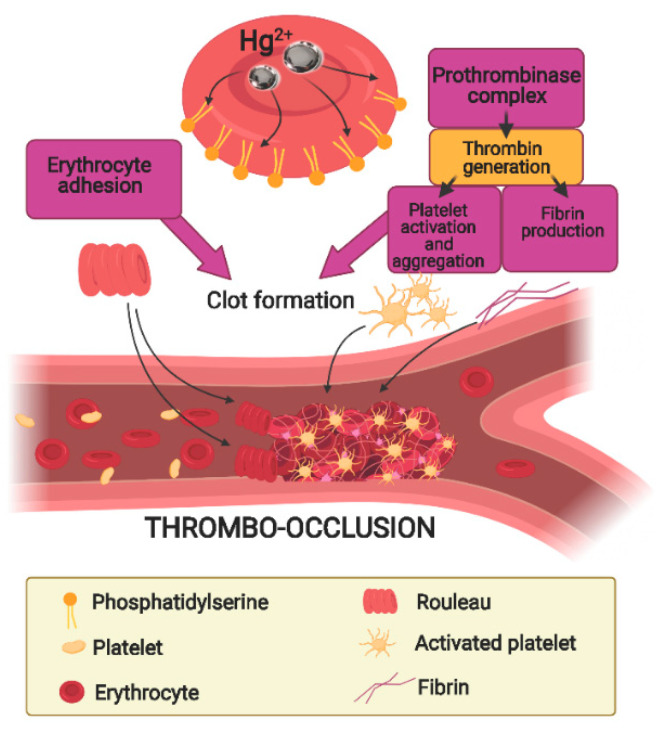
Hg-induced procoagulant activity in RBC. Hg-induced PS exposure on the outer surface of RBC enhances RBC adhesion to the endothelial cells. Moreover, PS-bearing RBC and MVs provide a site for assembling the prothrombinase complex, leading to thrombin generation and clot formation.

**Figure 4:**
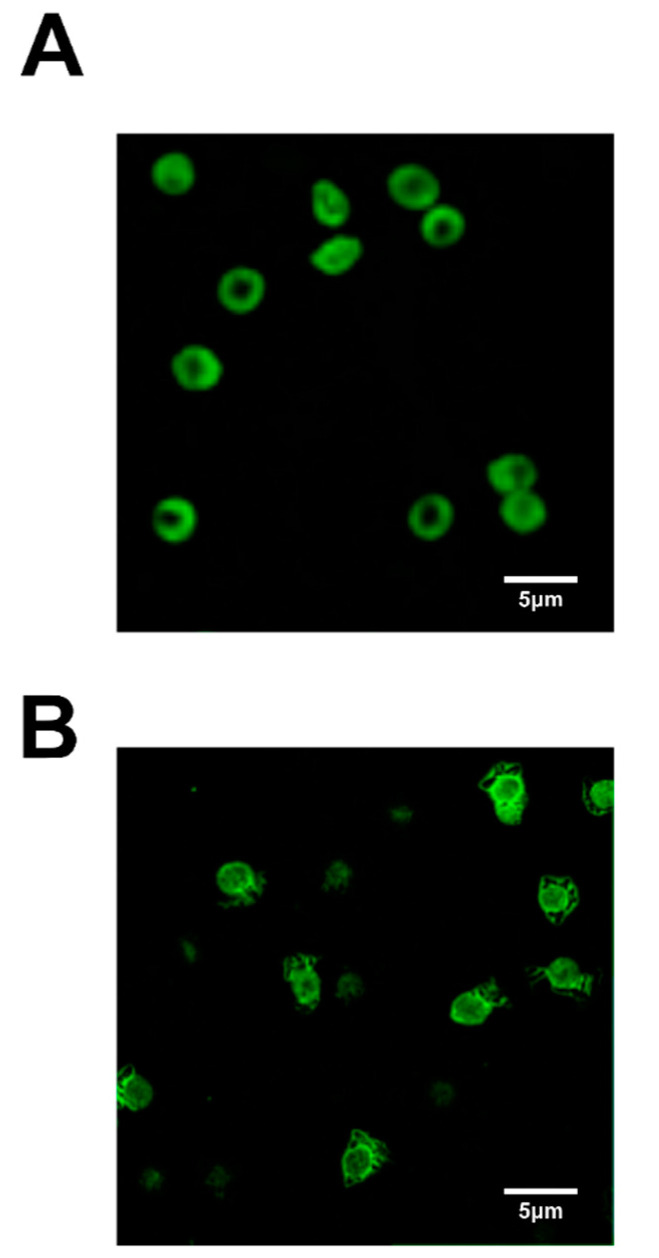
Morphological analysis of Hg-exposed RBC. Confocal Laser Scanning microscope imaging of intact RBC incubated in vitro in the presence of 20 μM HgCl_2_. At the end of incubation, cells were stained with Annexin V-FITC. (**A**) Untreated RBC; (**B**) HgCl_2_-treated RBC. Unpublished photos.

In recent years, particular attention has been focused on the mechanisms promoting vascular dysfunction and thrombotic events as a consequence of Hg-induced RBC damage (Figure 3). In this respect, workers occupationally exposed to Hg vapor show increased blood concentration of the heavy metal, associated with significant alterations in the coagulation system [120]. Among suggested mechanisms underlying the cardiovascular effects of Hg exposure, alterations of membrane asymmetry, due to PS exposure, enhances RBC adhesion to the endhothelial cells and clot formation. Hg-mediated SH depletion inhibits flippase and activates scramblase, increasing protein adhesion expression on the external surface of the cell and thrombin production [101]. In fact, PS-bearing RBC and MVs provide a binding site for assembling the prothrombinase complex, leading to thrombin generation, thus stimulating the coagulative cascade even at low-dose Hg^2+^ exposure. Alterations of RBC membrane proteins, leading to changes in cell shape and deformability, were also reported to be pro-thrombotic factors [121]. Changes in RBC shape have indeed been reported after in vitro exposure to this metal [121,122]. The underlying mechanisms are, also in this case, linked to the ability of this metal to bind SH groups. In fact, GSH and metallothionein, rich in free SH groups, have a pivotal role in the maintenance of erythrocyte integrity (e.g., the cytoskeleton) and ionic/energy balance (e.g., Na^2+^/K^+^-ATPase) with ATP depletion and increased calcium. These mechanisms lead to increased fragility of the RBC membrane and disruption and MVs liberation [101]. All together, these effects design RBC as an active contributor to the increase in thrombotic events associated with Hg intoxication (Figure 4) [121,123,124].

An additional key mechanism in which a role of RBC in the endothelial dysfunction has been suggested is NO production. Recent data point to RBC as key regulators of vasodilatation in peripheral tissue. These cells express the isoform eNOS and they display the ability to biosynthesize NO under oxygen deprivation, contributing to circulation homeostasis [125,126,127,128]. In this respect, RBC exposure to HgCl_2_ has been reported to decrease NOS activity, with consequent lowering of NO levels [78,129]. It is important to emphasise that the reported Hg-induced damage to hemoglobin (Hb) may also contribute to the decreased bioavailability of this key vasorelaxant molecule [130,131]. A tetrameric mercurized form of Hb, supposed to be dysfunctional in physiological conformational changes crucial for the oxygenation/deoxygenation process, can be observed after RBC exposure to Hg^2+^ [55]. As discussed in the introduction, accessible Cys residues represent the preferential site of Hg–protein interaction. It is worth noting, in this respect, that two critical Cys in position 93 of the beta-chains have been identified in Hb as NO ligand, playing a role in Hb-mediated NO release [130]. Consequently, the alteration of Cys-93, following Hg^2+^ interaction, might impair the Hb-mediated regulation of blood flow, therefore representing one of the physiologically important manifestations of Hg poisoning in these cells, thus in turn affecting the cardiovascular system. As a result, based on these findings, RBC acquired the title of surrogate model for the study of Hg-induced vascular dysfunction with full rights [129,132,133]. Finally, a different mechanism that may induce a decrease in NO availability is the generation of an oxidative microenvironment associated with Hg exposure. Indeed, it is well known that NO production usually decreases following endothelial dysfunction as well as under OS conditions [129,134]. In this context, when intact RBC are exposed in vitro to the presence of micromolar HgCl_2_ concentrations a dramatic increase in ROS generation is observed. ROS production is a late event and occurs subsequent to a decrease in the antioxidant thiol GSH, which significantly impairs the antioxidant defense system. In addition, an Hg-induced nitrosative stress is also reported [131].

## 7. Nutritional Aspects of Mercury Toxicity

Hg is a metal that is not essential for nutrition, in that it is not an essential component of our body and is not involved in any metabolic pathway. However, as discussed in the introduction, diet represents one of the most important routes of chronic exposure to Hg in general populations [135]. The greatest risk comes from eating contaminated food, especially fish, which accumulate high Hg content, due to its peculiar biogeochemical cycle. The critical point for fish contamination is Hg cycling in the Aquatic Ecosystems, where this metal is converted into its organic forms, mainly MeHg, by anaerobic microorganisms. This form, in turn, enters the aquatic trophic network via plankton and it tends to accumulate in biota in the passage through small fish, reaching its greatest concentration in carnivorous fish at the top of the food chain [36] due to the so-called process of biomagnification [136]. Importantly, only Hg biomagnifies in the food chain [137]. Following the water cycle, it pollutes surface and groundwater, involving crops, particularly cereals [138,139]. However, while there are foods responsible for Hg absorption in humans, on the other hand, there are foods containing bioactive compounds which are claimed to reduce its harmful health effects. Several naturally derived products have been tested as potential protective agents, either for prevention and/or as treatment against Hg-induced cytotoxicity [140]. In this context, dietary components capable of chelating heavy metals are increasingly being used [141,142] including dietary fibers able to perform Hg chelation during gastric-intestinal transit [143].

In recent years a possible utilization of garlic (*Allium sativium* L.) as an important detoxifying agent against heavy metal toxicity, including Hg, has been proposed [144]. This spice, indeed, widely used in preparing food as well as nutraceutical products, contains several organosulfur compounds, the most abundant being allicin [145] able to bind directly to Hg. Interestingly, garlic is also rich in selenium, an essential mineral, which hinders Hg toxicity by forming inactive selenium-Hg complexes as well as by preventing ROS-induced cellular damage. It is well known indeed that selenium strengthens the antioxidant defense system [146], being a co-factor of antioxidant enzymes such as glutathione peroxidase [147]. In this respect, as discussed in the introduction, since a key mechanism underlying heavy metal toxicity is the impairment of the antioxidant defense system, vitamin C and E have been tested as potential protective agents [148,149,150]. Furthermore, several phytochemicals with scavenging properties have been proved to actively counteract the heavy metal-induced body burden and cellular biochemical alterations [151], including curcumin [152,153] and epigallocatechin gallate [154].

Finally, several studies have revealed the role of olive oil polyphenols in the prevention of Hg toxicity. There is general agreement, based on a large body of converging evidence, that the antioxidant fraction, including polyphenols, significantly contributes to the health-promoting effect [155,156] of this typical lipidic source of the Mediterranean Diet, a dietary habit associated with a low incidence of several pathologies [157,158], including CVD [159]. Among these compounds, hydroxytyrosol (3,4-dihydroxyphenyl- ethanol; HT) is mainly responsible for the antioxidant properties of this food due to efficient scavenger activity [160]. Besides its well-recognized anti-inflammatory and anticancer properties [161,162], HT ameliorates the harmful effects of toxic agents, including acrylamide [163,164,165] and acrolein [166,167,168] as well as the mycotoxin ochratoxin-A [169]. Moreover, recent data indicate that this dietary component is able to counteract the toxic effects linked to exposure of heavy metals, including Hg. Mohan et al. reported that the ability of HT to promote the expression of nuclear factor erythroid 2-related factor 2 (Nrf2), which in turn elevates GSH levels, is crucial in ameliorating the neurotoxic effect of MeHg [170]. The protective effects of HT on the biochemical alterations induced in intact human RBC, subjected in vitro to treatment with HgCl_2_, has been recently investigated by our group [122,171]. The reported findings indicate that HT has the potential to modulate cytotoxicity and to counteract the OS induced in RBC by Hg treatment. An additional study also provides experimental evidence of the efficacy of HT in modulating the programmed suicidal death in these anucleate cells, also devoid of mitochondria and thus lacking any mitochondria-mediated apoptotic pathways. Cell preconditioning with an HT optimal dose, prior to exposure to HgCl_2_, causes a noteworthy decrease in PS-exposing RBC, almost restoring ATP and GSH content [117]. Also of clinical importance is the finding that HT prevents Hg-induced RBC morphological alterations which potentially enhance the procoagulant activity of these cells, resulting in a contributing factor to Hg-related thrombotic events. In recent years nutritional research as well as in vitro studies have focused on the effects of HT in the progression of atherosclerosis including the expression of adhesion molecules [172,173], a key mechanism implicated in plaque formation. Furthermore, HT inhibits in vitro LDL oxidation and counteracts the OS-induced endothelial dysfunction. The reported data on HT protective effects on Hg-induced RBC alterations reveal that prevention of metal toxicity should be regarded as an additional mechanism responsible for the health-promoting potential of this dietary phenol on the cardiovascular system.

## 8. Conclusions

Human exposure to Hg, in its elementary and molecular forms, may cause severe damage to the entire organism. If massive exposure is a rare event, mostly related to accidents or occupational exposure, chronic intoxication, due to low-level Hg exposure, is becoming a growing insidious concern for a larger portion of the population. In this respect, the pro-oxidant effect of this metal even at low-dose exposure has shifted the focus from a CNS-centred view to a more holistic view of Hg-induced health damage.

In this context, the data discussed in this review point to the involvement of the cardiovascular system as a major target of long-term toxicity, ranging from endothelial dysfunction to pro-coagulative transformation of RBC. Taken together, these effects expose all organs and systems to increased thrombotic risk. Accordingly, anti-vascular endothelial growth factor) monoclonal antibodies have been proposed, in preclinical settings based on the upregulation of these mediators in Hg-induced endothelial dysfunctions. Moreover, the data on the morphological and metabolic alteration in RBC induced by Hg point to the key role of RBC in Hg-related endothelial dysfunction and let us propose potential alternative targets for the prevention of systemic damage induced by this heavy metal.

Chelation therapy remains the therapy of choice in massive exposure to heavy metals, including Hg. The rationale for using thiol-based chelators lies in their ability to form a ring-like complex with the metal, facilitating its renal excretion. Chelation therapy, however, is not without risks since chelator agents are endowed with a degree of toxicity. In addition, the accurate choice of dosage and timing of administration is essential to avoid depletion of essential metals, with consequent worsening of the general condition of the patient.

The possibility of reducing heavy metal toxicity through bioactive dietary components has attracted great interest recently. Since contaminated food is the major source of chronic Hg exposure in the general population, fighting it on the same battlefield is an intriguing challenge. In addition to its primary role of providing adequate quantities of nutrients, a healthy diet is a vital factor in reducing morbidity and mortality from chronic diseases. In this context, fruit and vegetables contain thousands of phytochemicals, endowed with a variety of biological and pharmacological activities, which are able to strengthen biological functions with the aim of promoting human health and reducing the risk of disease. As far as metal toxicity is concerned, nutritional components, including vitamin C and E and selenium have been proved to be protective, representing an attractive tool to help our body to fight off the adverse effects of heavy metals. Moreover, data discussed in this review provide experimental evidence that antioxidant polyphenols, normally present in our diet, especially the Mediterranean, have the potential to modulate Hg toxicity, therefore representing ideal candidates for nutritional/nutraceutical strategies to counteract the clinical outcomes of chronic Hg exposure in humans, particularly related to CVD.

## Abbreviations

ACE	Angiotensin-converting enzyme
COMT	Catecholamine-O-methyl transferase
CVD	Cardiovascular diseases
Cys	Cysteine
GSH	Glutathione
Hb	Hemoglobin
Hg	Mercury
Hg^0^	Elemental mercury
Hg^2+^	Mercuric
HT	Hydroxythyrosol
MeHg	Methylmercury
MVs	Microvesicles
NO	Nitric oxide
NOS	Nitric oxide synthase
Nrf2	Nuclear factor erythroid 2-related factor 2
OS	Oxidative stress
PS	Phosphatidylserine
RBC	Erythrocytes
ROS	Reactive oxygen species
SH	Sulfhydryl
